# Evaluation of the long-term prognostic ability of triglyceride-glucose index for elderly acute coronary syndrome patients: a cohort study

**DOI:** 10.1186/s12933-021-01443-y

**Published:** 2022-01-06

**Authors:** Yang Jiao, Yongkang Su, Jian Shen, Xiaoling Hou, Ying Li, Jihang Wang, Bing Liu, Dongfeng Qiu, Zhijun Sun, Yundai Chen, Qing Xi, Mingzhi Shen, Zhenhong Fu

**Affiliations:** 1 Senior Department of Cardiology, The Sixth Medical Center, Chinese PLA General Hospital and Chinese PLA Medical School, Beijing, 100853 China; 2 Department of Geriatrics, The Second Medical Center, Chinese PLA General Hospital, Beijing, 100853 China; 3 Department of Cardiology, Hainan Hospital, Chinese PLA General Hospital, Sanya, 572000 Hainan China; 4 Department of Cardiology, 970 Hospital, Chinese PLA Joint Logistic Support Force, Weihai, 264200 Shandong China; 5 The First Medical Center, Chinese PLA General Hospital, Beijing, 100853 China

**Keywords:** Triglyceride glucose index, Elderly patient, Acute coronary syndrome, Insulin resistance, Prognosis

## Abstract

**Background:**

With the advancement of the world population aging, more attention should be paid to the prognosis of elderly patients with acute coronary syndrome (ACS). Triglyceride-glucose (TyG) index is a reliable indicator of insulin resistance (IR) and is closely related to traditional risk factors of cardiovascular disease (CVD). However, the effect of TyG index on the prognosis of long-term adverse events in elderly ACS patients has not been reported. This study evaluated the prognostic power of TyG index in predicting adverse events in elderly ACS patients.

**Methods:**

In this study, 662 ACS patients > 80 years old who were hospitalized from January 2006 to December 2012 were enrolled consecutively and the general clinical data and baseline blood biochemical indicators were collected. The follow-up time after discharge was 40–120 months (median, 63 months; interquartile range, 51‒74 months). In addition, the following formula was used to calculate the TyG index: Ln [fasting TG (mg/dL) × FBG (mg/dL)/2], and patients were divided into three groups according to the tertile of the TyG index.

**Results:**

The mean age of the subjects was 81.87 ± 2.14 years, the proportion of females was 28.10%, and the mean TyG index was 8.76 ± 0.72. The TyG index was closely associated with the traditional risk factors of CVD. In the fully-adjusted Cox regression model, the Hazard ratio (95% CI) of all-cause mortality (in tertile 3) was 1.64 (1.06, 2.54) and major adverse cardiac event (MACE) (in tertile 3) was 1.36 (1.05, 1.95) for each SD increase in the TyG index. The subgroup analyses also confirmed the significant association of the TyG index and long-term prognosis.

**Conclusion:**

The TyG index is an independent predictor of long-term all-cause mortality and MACE in elderly ACS patients.

## Background

Coronary heart disease (CHD) is the number one killer threatening human health, and the incidence increases significantly with age [1]. With the aggravation of population aging, the proportion of elderly patients hospitalized for acute coronary syndrome (ACS) is increasing, and studies showed that more than 30% of patients are over 75 years old [2, 3]. The risk of bleeding and acute myocardial infarction post-percutaneous coronary intervention (re-MI) at 1-year follow-up is more than doubled in elderly patients [4]. Advanced aging has a profound impact on the health of the elderly. With the increase of age, changes in cardiovascular structure and function accelerate the progression of CHD, and elderly patients have more severe lesions [5]. Insulin resistance (IR) is the main pathogenesis of diabetes mellitus (DM) and is a common marker of systemic inflammatory response and metabolic disorders, which is closely related to the progression of coronary atherosclerosis [6–8]. However, IR was not a traditional risk factor of CHD [9]. Multiple studies have shown that the TyG index is associated with multiple risk factors of CHD, such as hypertension, diabetes, obesity and metabolic syndrome, and it also can predict the prognosis of patients with CHD and in-stent restenosis (ISR) [10–15]. However, among the clinical studies on the prognosis of ACS patients, there are few studies on the predictive value of the TyG index, especially with regard to elderly people (who have not been clearly reported), and the pathophysiological mechanism has not been clarified. In this study, we analyzed the TyG index after admission and the incidence of main cardiovascular adverse events within 10 years after discharge of elderly ACS patients, aiming to investigate whether the TyG index is an independent risk factor for the long-term prognosis of elderly ACS patients.

## Methods

### Study population

A total of 720 patients aged 80 years and above who were hospitalized for coronary angiography due to ACS symptoms in the Cardiology Department of Chinese People’s Liberation Army (PLA) General Hospital from January 2006 to December 2012 were enrolled. Of these patients, 699 provided informed consent and were included in the study. During the follow-up period, 37 were lost to follow up, and 662 were finally available for our statistical analysis. The follow-up rate was 94.7%. The main exclusion criteria were as follows: (1) patients with severe valvular heart disease, pulmonary hypertension, severe liver insufficiency, rheumatoid arthritis, malignant tumors, infectious diseases, or body mass index (BMI) ≥ 45 kg/m^2^; (2) patients with familial hypertriglyceridemia (triglyceride ≥ 5.65 mmol/L); and (3) patients with neuropsychiatric disorders that prevented them from cooperating with the researcher. Most importantly, our study was performed in line with the Declaration of Helsinki and was approved by Ethics Service Center of Chinese PLA General Hospital, and all patients signed the informed consent.

### Intervention and management

Coronary intervention and periprocedural management were performed according to current guidelines in the Cardiac Interventional Center of PLA General Hospital to confirm the diagnosis of CHD, and all angiography results were analyzed using the same image analysis software. Loading doses of aspirin (300 mg) and clopidogrel (300 mg) were given before the intervention. The severity of coronary artery stenosis was recorded by Gensini score, and the experimental data recorders passed uniform professional training. According to the results of coronary angiography, patients were given individualized interventions, including intensive treatment with medicine, percutaneous coronary intervention (PCI) or coronary artery bypass grafting (CABG), and long-term follow-up observation was conducted after discharge.

### Data collection and definitions

We recorded general information (age, gender, BMI, heart rate, blood pressure, left ventricular ejection fraction (LVEF), and Gensini score), cardiovascular risk factors (hypertension, diabetes, hyperlipidemia, previous myocardial infarction (MI), previous stroke, chronic kidney injury (CKD) and current smoking), fasting blood biochemical indicators at 6 am the next day after admission (total cholesterol (TC), triglyceride (TG), low-density lipoprotein-cholesterol (LDL-C), high-density lipoprotein-cholesterol (HDL-C), the estimated glomerular filtration rate (eGFR), fasting blood glucose (FBG), uric acid (UA), calculated TyG index), cardiovascular medication experience (aspirin, clopidogrel, statins, β-blockers, angiotensin-converting enzyme inhibitor (ACEI)/angiotensin receptor blocker (ARB)), culprit artery (left anterior descending artery (LAD), left circumflex artery (LCX), left main coronary artery (LM), right coronary artery (RCA), and multivessel lesion), and treatment strategies (intensive treatment with medicine, PCI and CABG).

The TyG index was calculated by the following formula: Ln [fasting TG (mg/dL) × FBG (mg/dL)/2] [16]. The diagnosis of T_2_DM included: (1) FPG ≥ 7.0 mmol/L, and/or random blood glucose (RBG) ≥ 11.1 mmol/L, and/or 2 h plasma glucose after oral glucose tolerance test (OGTT) ≥ 11.1 mmol/L [17]. Hyperlipidemia was defined as: ICD-10 code E78 with lipid-lowering agents or serum total cholesterol ≥ 240 mg/dL [16].

The estimated glomerular filtration rate (eGFR) was calculated by the Chinese modified Modification of Diet in Renal Disease equation [18]:

$${\text{eGFR}}\,\left( {{\text{mL/min/1}}{.73}\,{\text{m}}^{{2}} } \right) = 175 \times {\text{standardized}}\,{\text{creatinine}}\,\left( {\text{mg/dL}} \right)^{ - 1.234} \times {\text{age}}\,\left( {{\text{year}}} \right)^{ - 0.179} \times 0.79\,({\text{if female}}).$$

Standardized creatinine (Scr) was calculated by the calibration equation [19]:

$${\text{Scr}}\,\left( {\text{mg/dL}} \right) = 0.795 \times \left[ {enzymatic\,method\,Scr\left( {\text{mg/dL}} \right)} \right] + 0.29.$$

Chronic Kidney Disease was defined as eGFR < 60 mL/min/1.73 m^2^.

Based on coronary angiography results, the multivessel lesion was defined as the number of culprit vessels with significantly diameter stenosis ≥ 50% was more than 2.

### Endpoints and follow-up

A clinical follow-up was performed once every 12 months after discharge via family interview, telephone record or medical records in the event of outcomes, and the follow-up period lasted up to 10 years. The main outcomes of our study were the occurrence of major adverse cardiac event (MACE), including nonfatal AMI, coronary artery revascularization (PCI or CABG) and all-cause mortality (cardiac or non-cardiac mortalities).

### Statistical analysis

Distributions of characteristics of the participants were illustrated according to the TyG index tertiles. The measurement data of normal distribution were expressed as mean ± standard deviation (SD), and if the variances were homogeneous, the t-test was used; if the variances were not homogeneous, the rank-sum test was used. The measurement data of non-normal distribution were represented by medians with interquartile range (IQR). The enumeration data were expressed numerically and differences between groups were assessed using the chi-square test. Analysis of variance was used to compare data between groups. In addition, the associations between the TyG index and clinical parameters were assessed by using Pearson’s correlation test. Unadjusted survival curves were generated in Kaplan–Meier plots with log-rank tests.

Univariate Cox regression analysis (Hazard ratio [HR], 95% CI) was used to identify the factors associated with all-cause mortality and MACE. P < 0.05 meant the statistical significance of the difference. We used Cox proportional hazards models to estimate the association between TyG index and all-cause mortality and MACE. Three regression models were built and potential confounders were adjusted in these models. Model 1 was the unadjusted model, model 2 was the partially adjusted model that was controlled for age and sex, and model 3 was the fully adjusted model that was controlled for variables in model 2 plus BMI, SBP, DBP, LVEF, Gensini score, hypertension, diabetes, hyperlipidemia, previous MI, previous stroke, CKD, current smoking, TC, LDL-C, HDL-C, eGFR, UA, aspirin, clopidogrel, statin, β-blocker, ACEI/ARB, LM lesion, multivessel lesion and treatment.

The TyG index was transformed into three categorical variables for the primary analysis. For the trend test, the newly categorical variable was also recoded a continuous variable and entered into the regression models. We also standardized the TyG index, then put it into the regression models to determine the relationships between the increase of TyG per SD and endpoints.

Additionally, we conducted subgroup analyses to explore whether the associations between the TyG index and all-cause mortality and MACE was modified by the following variables: sex, hypertension, diabetes, previous stroke, previous MI, hyperlipidemia, CKD, current smoking, BMI and multivessel lesion. Interactions between the TyG index and each of the above variables were tested. Findings are reported by hazard ratios (HR) and 95% confidence intervals (CI).

Two-sided P < 0.05 was considered statistically significant. All data in this study were processed using SPSS software Version 23.0 (IBM Corporation, Armonk, NY, USA), the statistical software packages R (http://www.R-project.org, The R Foundation) and Empower Stats (http://www.empowerstats.com, X&Y Solutions, Inc., Boston, MA).

## Results

### Baseline characteristics of study participants by TyG index

A total of 662 elderly patients with ACS were enrolled in this study, with an average age of 81.87 ± 2.14 years, the female proportion was 28.10%, and the average TyG index was 8.76 ± 0.72. Patients were divided into three groups according to the tertile of TyG index. Participants with higher TyG index were more likely to have elevated BMI, HR, TC, TG, LDL-C, FBG, UA, GENSINI score, and lower height, LVEF, HDL-C, and eGFR. In the high TyG index group, the proportion of patients with female, hypertension, DM, hyperlipidemia, previous stroke, and multivessel lesion were likely to be higher (Table 1).

**Table 1:** Baseline characteristics of patients stratified by tertile of TyG index

Variable	Total	Tertile 1	Tertile 2	Tertile 3	P-value
N	662	221	221	220	–
General conditions					
Age, years	81.87 ± 2.14	81.88 ± 2.04	82.15 ± 2.38	81.58 ± 1.94	0.062
Female, n (%)	186 (28.10%)	35 (15.84%)	61 (27.60%)	90 (40.91%)	** < 0.001**
Height, cm	165.32 ± 8.25	166.36 ± 7.50	165.32 ± 8.72	164.28 ± 8.39	**0.025**
Weight, kg	67.20 ± 10.68	66.52 ± 10.73	67.31 ± 10.26	67.76 ± 11.06	0.463
BMI, kg/m^2^	24.57 ± 3.40	24.01 ± 3.40	24.62 ± 3.30	25.07 ± 3.43	**0.004**
HR, beat/min	74.81 ± 14.02	73.15 ± 13.54	74.13 ± 12.27	77.15 ± 15.79	**0.008**
SBP, mmHg	137.08 ± 21.79	136.85 ± 22.00	137.33 ± 21.04	137.06 ± 22.40	0.902
DBP, mmHg	71.44 ± 12.12	71.96 ± 11.83	71.19 ± 11.94	71.15 ± 12.60	0.731
LVEF, %	55.65 ± 9.91	57.06 ± 9.42	55.08 ± 10.12	54.82 ± 10.06	**0.034**
Gensini score	53.65 ± 42.65	40.79 ± 34.51	59.78 ± 41.27	60.41 ± 48.29	** < 0.001**
Risk factors, n (%)					
Hypertension	511 (77.19%)	158 (71.49%)	173 (78.28%)	180 (81.82%)	**0.032**
Diabetes	231 (34.89%)	46 (20.81%)	68 (30.77%)	117 (53.18%)	** < 0.001**
Hyperlipidemia	151 (22.81%)	36 (16.29%)	54(24.43%)	61 (27.73%)	**0.011**
Previous MI	120 (18.13%)	34 (15.38%)	44 (19.91%)	42 (19.09%)	0.421
Previous stroke	138 (20.85%)	43 (19.46%)	46 (20.81%)	49 (22.27%)	**0.042**
CKD	78 (11.78%)	19 (8.60%)	29 (13.12%)	30 (13.64%)	0.195
Current smoking	164 (24.77%)	67 (30.32%)	47 (21.27%)	50 (22.73%)	0.061
Baseline blood features					
TC, mmol/L	4.11 ± 0.97	3.87 ± 0.89	4.10 ± 0.91	4.36 ± 1.04	** < 0.001**
TG, mmol/L	1.39 ± 0.72	0.84 ± 0.22	1.28 ± 0.33	2.04 ± 0.82	** < 0.001**
LDL-C, mmol/L	2.36 ± 0.84	2.19 ± 0.75	2.38 ± 0.82	2.52 ± 0.91	** < 0.001**
HDL-C, mmol/L	1.13 ± 0.36	1.23 ± 0.40	1.13 ± 0.36	1.01 ± 0.28	** < 0.001**
eGFR, mL/min/1.73 m^2^	70.85 ± 23.11	73.69 ± 17.27	69.93 ± 19.77	68.93 ± 30.11	**0.003**
FBG, mmol/L	7.05 ± 4.13	5.32 ± 1.17	6.42 ± 1.75	9.40 ± 6.17	** < 0.001**
UA, umol/L	351.59 ± 149.66	335.69 ± 200.17	356.29 ± 103.22	362.85 ± 127.46	**0.001**
TyG index	8.76 ± 0.72	8.08 ± 0.61	8.72 ± 0.15	9.47 ± 0.42	** < 0.001**
Cardiovascular medications, n (%)					
Aspirin	642 (96.98%)	210 (95.02%)	217 (98.19%)	215 (97.73%)	0.110
Clopidogrel	632 (95.47%)	208 (94.12%)	216 (97.74%)	208 (94.55%)	0.136
Statin	615 (92.90%)	206 (93.21%)	203 (91.86%)	206 (93.64%)	0.748
β-blocker	418 (63.14%)	122 (55.20%)	147 (66.52%)	149 (67.73%)	**0.011**
ACEI/ARB	364 (54.98%)	107 (48.42%)	126 (57.01%)	124 (59.55%)	**0.040**
Angiography, n (%)					
LAD lesion	560 (84.59%)	184 (83.26%)	192 (86.88%)	184 (83.64%)	0.511
LCX lesion	383 (57.85%)	129 (58.37%)	128 (57.92%)	126 (57.27%)	0.601
RCA lesion	429 (64.80%)	144 (65.16%)	137 (61.99%)	148 (67.27%)	0.251
LM lesion	109 (16.47%)	26 (11.76%)	39 (17.65%)	44 (20.00%)	0.056
Multivessel lesion	466 (70.39%)	138 (62.44%)	164 (74.21%)	164 (74.55%)	**0.007**
Treatment, n (%)					0.081
Intensive medication	241 (36.40%)	94 (42.53%)	71 (32.13%)	76 (34.55%)	
PCI	405 (61.18%)	125 (56.56%)	142 (64.25%)	138 (62.73%)	
CABG	16 (2.42%)	2 (0.90%)	8 (3.62%)	6 (2.73%)	

Data are shown as mean ± standard deviation (SD) or number (*n*) of patients. ^△^P < 0.05, ^△△^P < 0.01, Tertile 1 vs. Tertile 2; ^#^P < 0.05, ^##^P < 0.01, Tertile 1 vs. Tertile 3; ^*^P < 0.05, ^**^P < 0.01, Tertile 2 vs. Tertile 3. P values in bold are < 0.05

BMI, body mass index; HR heart rate; SBP, systolic blood pressure; DBP, diastolic blood pressure; LVEF, left ventricular ejection fraction; MI, myocardial infarction; CKD, chronic kidney disease; TC, total cholesterol; TG, triglyceride; LDL-C, low-density lipoprotein-C; HDL-C, high-density lipoprotein-C; eGFR, estimated glomerular filtration rate; FBG, fasting blood glucose; UA, uric acid; TyG, triglyceride glucose; ACEI, angiotensin-converting enzyme inhibitor; ARB, angiotensin receptor blocker; LAD, left anterior descending artery; LCX, left circumflex artery; RCA, right coronary artery; LM, left main coronary artery; PCI, percutaneous coronary intervention; CABG, coronary artery bypass grafting

### Correlations between TyG index and clinical variables

In order to identify the association between the TyG index and risk factors, Spearman’s rank or Pearson’s correlation analysis was performed. As shown in Table 2, the TyG index was positively correlated with BMI, Gensini score, TC, TG, LDL-C, FBG and UA, and negatively correlated with DBP, LVEF, HDL-C. Except for TC and FBG, the TyG index had the highest positive correlation with Gensini score and the highest negative correlation with HDL-C.

**Table 2:** Correlations between TyG index and traditional cardiovascular risk factors

Variable	Correlation coefficient	P value
Age	− 0.0672	0.0841
BMI	0.0920	**0.0179**
SBP	− 0.0491	0.2068
DBP	− 0.1049	**0.0069**
LVEF	− 0.0778	**0.0453**
Gensini score	0.1963	** < 0.001**
TC	0.1742	** < 0.001**
TG	0.7982	** < 0.001**
LDL-C	0.1151	**0.0030**
HDL-C	− 0.2370	** < 0.001**
eGFR	− 0.0599	0.1234
FBG	0.5524	** < 0.001**
UA	0.0804	**0.0386**

P values in bold are < 0.05

BMI, body mass index; SBP, systolic blood pressure; DBP, diastolic blood pressure; LVEF, left ventricular ejection fraction; TC, total cholesterol; TG, triglyceride; LDL-C, low-density lipoprotein-C; HDL-C, high-density lipoprotein-C; eGFR, estimated glomerular filtration rate; FBG, fasting blood glucose; UA, uric acid

### Univariate regression analysis of long-term prognostic correlation among three groups

The follow-up time of this study was 40–120 months (median, 63 months; interquartile range, 51‒74 months), and the main outcomes were all-cause mortality and MACE. All-cause mortality and MACE of the three groups were analyzed by univariate Cox regression. As presented in Table 3, that age, LVEF, Gensini score, DM, previous stroke, CKD, TC, TG, LDL-C, eGFR, FBG, TyG index, aspirin, LM lesion, and multivessel lesion were found to be independent risk factors for all-cause mortality in ACS patients. Meanwhile, SBP, LVEF, Gensini score, DM, previous stroke, CKD, TC, TG, eGFR, FBG, TyG index, aspirin, LM lesion, and multivessel lesions were found to be independent risk factors for MACE in ACS patients.

**Table 3:** Results of univariate Cox regression analysis

Variable	All-cause mortality		MACE	
	HR (95% CI)	P value	HR (95% CI)	P value
Age	1.08 (1.02, 1.15)	**0.0117**	1.03 (0.98, 1.09)	0.2401
Male	1.05 (0.77, 1.43)	0.7774	1.15 (0.88, 1.51)	0.3181
BMI	0.96 (0.92, 1.00)	0.0839	0.98 (0.95, 1.02)	0.3472
SBP	0.99 (0.99, 1.00)	0.0581	0.99 (0.99, 1.00)	**0.0105**
DBP	0.99 (0.98, 1.01)	0.3366	1.00 (0.99, 1.01)	0.5432
LVEF	0.96 (0.95, 0.98)	** < 0.0001**	0.97 (0.96, 0.99)	** < 0.0001**
Gensini score	1.01 (1.00, 1.01)	** < 0.0001**	1.01 (1.00, 1.01)	** < 0.0001**
Hypertension	1.07 (0.76, 1.49)	0.7124	0.93 (0.71, 1.24)	0.6376
Diabetes	1.46 (1.10, 1.93)	**0.0084**	1.41 (1.11, 1.79)	**0.0050**
Hyperlipidemia	0.81 (0.57, 1.15)	0.2304	0.94 (0.71, 1.26)	0.6878
Previous MI	1.05 (0.75, 1.49)	0.7642	1.14 (0.85, 1.52)	0.3852
Previous stroke	1.50 (1.09, 2.04)	**0.0116**	1.54 (1.18, 2.01)	**0.0014**
CKD	2.30 (1.62, 3.25)	** < 0.0001**	1.81 (1.31, 2.48)	**0.0003**
Current smoking	1.08 (0.78, 1.48)	0.648	1.10 (0.84, 1.44)	0.4967
TC	1.26 (1.09, 1.45)	**0.0015**	1.14 (1.01, 1.29)	**0.0295**
TG	1.22 (1.03, 1.44)	**0.0190**	1.27 (1.09, 1.48)	**0.0018**
LDL-C	1.25 (1.06, 1.48)	**0.0074**	1.14 (0.99, 1.31)	0.0738
HDL-C	0.68 (0.44, 1.04)	0.0783	0.70 (0.49, 1.01)	0.0594
eGFR	0.98 (0.97, 0.98)	** < 0.0001**	0.99 (0.98, 0.99)	** < 0.0001**
FBG	1.05 (1.03, 1.06)	** < 0.0001**	1.04 (1.02, 1.06)	** < 0.0001**
UA	1.00 (1.00, 1.00)	**0.0035**	1.00 (1.00, 1.00)	**0.0160**
TyG index	1.75 (1.42, 2.16)	** < 0.0001**	1.60 (1.32, 1.92)	** < 0.0001**
Aspirin	0.38 (0.21, 0.70)	**0.0019**	0.43 (0.25, 0.75)	**0.0031**
Clopidogrel	0.60 (0.34, 1.05)	0.0711	0.63 (0.39, 1.03)	0.0653
Statin	0.98 (0.59, 1.64)	0.9511	1.40 (0.86, 2.30)	0.1785
β-blocker	1.06 (0.80, 1.42)	0.6721	1.23 (0.96, 1.59)	0.1021
ACEI/ARB	1.11 (0.84, 1.48)	0.4501	1.13 (0.89, 1.43)	0.3199
LM lesion	1.53 (1.09, 2.14)	**0.0141**	1.78 (1.34, 2.35)	** < 0.0001**
Multivessel lesion	1.44 (1.04, 2.00)	**0.0299**	1.62 (1.22, 2.15)	**0.0009**

P values in bold are < 0.05

BMI, body mass index; SBP, systolic blood pressure; DBP, diastolic blood pressure; LVEF, left ventricular ejection fraction; MI, myocardial infarction; CKD, chronic kidney disease; TC, total cholesterol; TG, triglyceride; LDL-C, low-density lipoprotein-C; HDL-C, high-density lipoprotein-C; eGFR, estimated glomerular filtration rate; FBG, fasting blood glucose; UA, uric acid; TyG, triglyceride glucose; ACEI, angiotensin-converting enzyme inhibitor; ARB, angiotensin receptor blocker; LM, left main coronary artery

### Association between TyG index and long-term prognosis

During the follow-up period, a total of 277 MACEs occurred and 201 of these were attributed to all-cause mortality. As shown in Table 4, for each SD increase in the TyG index, the HR (95% CI) of all-cause mortality was 1.28 (1.06, 1.56) in the fully adjusted regression model. The fully adjusted HRs (95%CIs) for all-cause mortality for subjects in tertile 2 and tertile 3 were 1.18 (0.79, 1.79) and 1.64 (1.06, 2.54), respectively. The increased risk of all-cause mortality from tertile 1 to tertile 3 was statistically significant (P for trend = 0.0204).

**Table 4:** Multivariable Cox regression analyses for the association between TyG index and all-cause mortality

TyG index	Events (No.), n (%)	HR (95% CI)		
		Model 1	Model 2	Model 3
Per SD increase	201 (30.36)	1.50 (1.29, 1.73)***	1.56 (1.34, 1.81)***	1.28 (1.06, 1.56)*
Tertile 1	44 (19.91)	1 (Reference)	1 (Reference)	1 (Reference)
Tertile 2	64 (28.96)	1.42 (0.96, 2.08)	1.45 (0.98, 2.12)	1.18 (0.79, 1.79)
Tertile 3	93 (42.27)	2.32 (1.62, 3.33)***	2.54 (1.76, 3.67)***	1.64 (1.06, 2.54)*
P for trend		< 0.001	< 0.001	0.0204

Model 1: unadjusted

Model 2: adjusted for age and sex

Model 3: adjusted for age, gender, BMI, SBP, DBP, LVEF, Gensini score, hypertension, diabetes, hyperlipidemia, previous MI, previous stroke, CKD, current smoking, TC, LDL-C, HDL-C, eGFR, UA, aspirin, clopidogrel, statin, β-blocker, ACEI/ARB, LM lesion, multivessel lesion and treatment

*P < 0.05

**P < 0.01

***P < 0.001

Similarly, the significant association between the TyG index and MACE was also found in the fully adjusted regression model (Table 5). For each SD increase in the TyG index, the HR (95% CI) of MACE rate was 1.21 (1.02, 1.43) in the fully adjusted regression model. The fully adjusted HR (95%CIs) for MACE in tertile 3 was 1.36 (1.05, 1.95). The increased risk of MACE from tertile 1 to tertile 3 was statistically significant (P for trend = 0.0214).

**Table 5:** Multivariable Cox regression analyses for the association between TyG index and MACE

TyG index	Events (No.), n (%)	HR (95% CI)		
		Model 1	Model 2	Model 3
Per SD increase	277 (41.84)	1.40 (1.22, 1.60)***	1.47 (1.28, 1.69)***	1.21 (1.02, 1.43)*
Tertile 1	70 (31.67)	1 (Reference)	1 (Reference)	1 (Reference)
Tertile 2	92 (41.63)	1.31 (0.96, 1.79)	1.34 (0.98, 1.83)	1.09 (0.78, 1.52)
Tertile 3	115 (52.27)	1.86 (1.38, 2.50)***	2.02 (1.49, 2.74)***	1.36 (1.05, 1.95)*
P for trend		< 0.001	< 0.001	0.0214

Model 1: unadjusted

Model 2: adjusted for age and sex

Model 3: adjusted for age, gender, BMI, SBP, DBP, LVEF, Gensini score, hypertension, diabetes, hyperlipidemia, previous MI, previous stroke, CKD, current smoking, TC, LDL-C, HDL-C, eGFR, UA, aspirin, clopidogrel, statin, β-blocker, ACEI/ARB, LM lesion, multivessel lesion and treatment

*P < 0.05

**P < 0.01

***P < 0.001

**Fig. 1:**
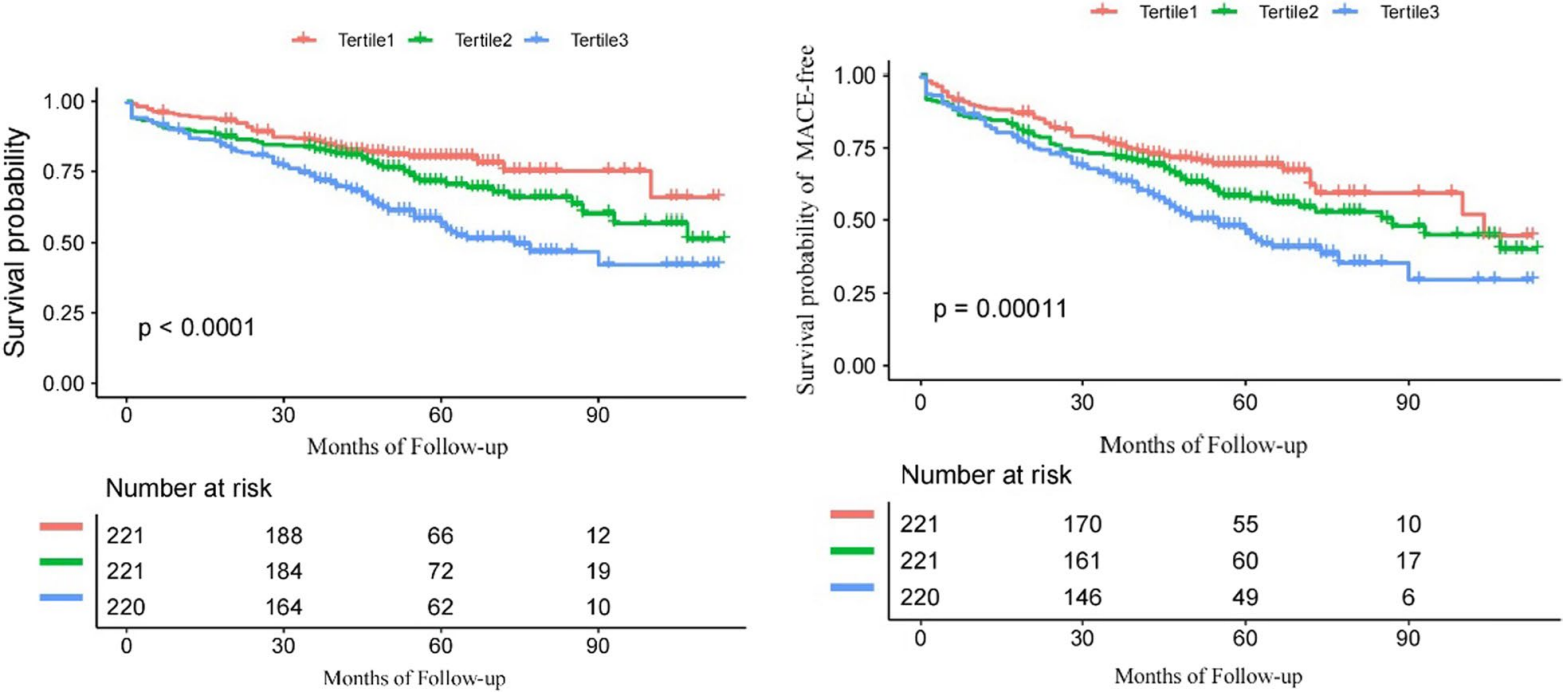
Kaplan–Meier survival curves of survival probability (**A**) and survival probability of MACE-free (**B**) for the elderly ACS patients

Kaplan Meier curves of the long-term survival and survival of MACE-free in elderly ACS patients for the TyG index tertiles are presented in Fig. 1. The survival probability and the survival probability of MACE-free in the tertile 1 group was significantly higher than that in the tertiel 3 group (P < 0.001). The cumulative incidence of all-cause death and MACE increased with higher tertiles of the TyG index.

### Subgroup analyses for association between TyG index and long-term prognosis

**Fig. 2:**
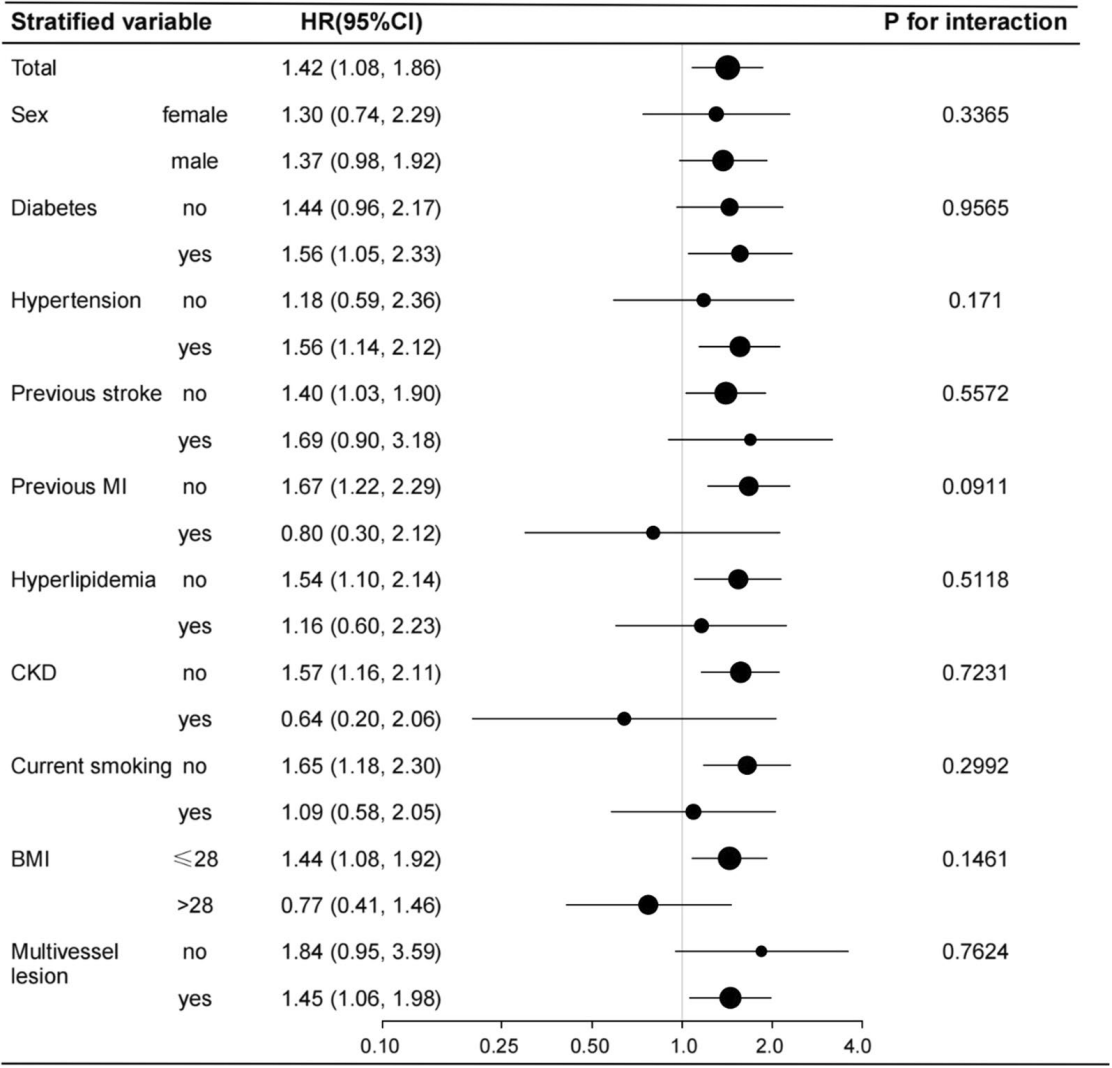
Forest plot investigating the association between the TyG index and all-cause mortality in different subgroups. Adjusted for age, gender, BMI, SBP, DBP, LVEF, Gensini score, hypertension, diabetes, hyperlipidemia, previous MI, previous stroke, CKD, current smoking, TC, LDL-C, HDL-C, eGFR, UA, aspirin, clopidogrel, statin, β-blocker, ACEI/ARB, LM lesion, multivessel lesion except for the stratified variable

**Fig. 3:**
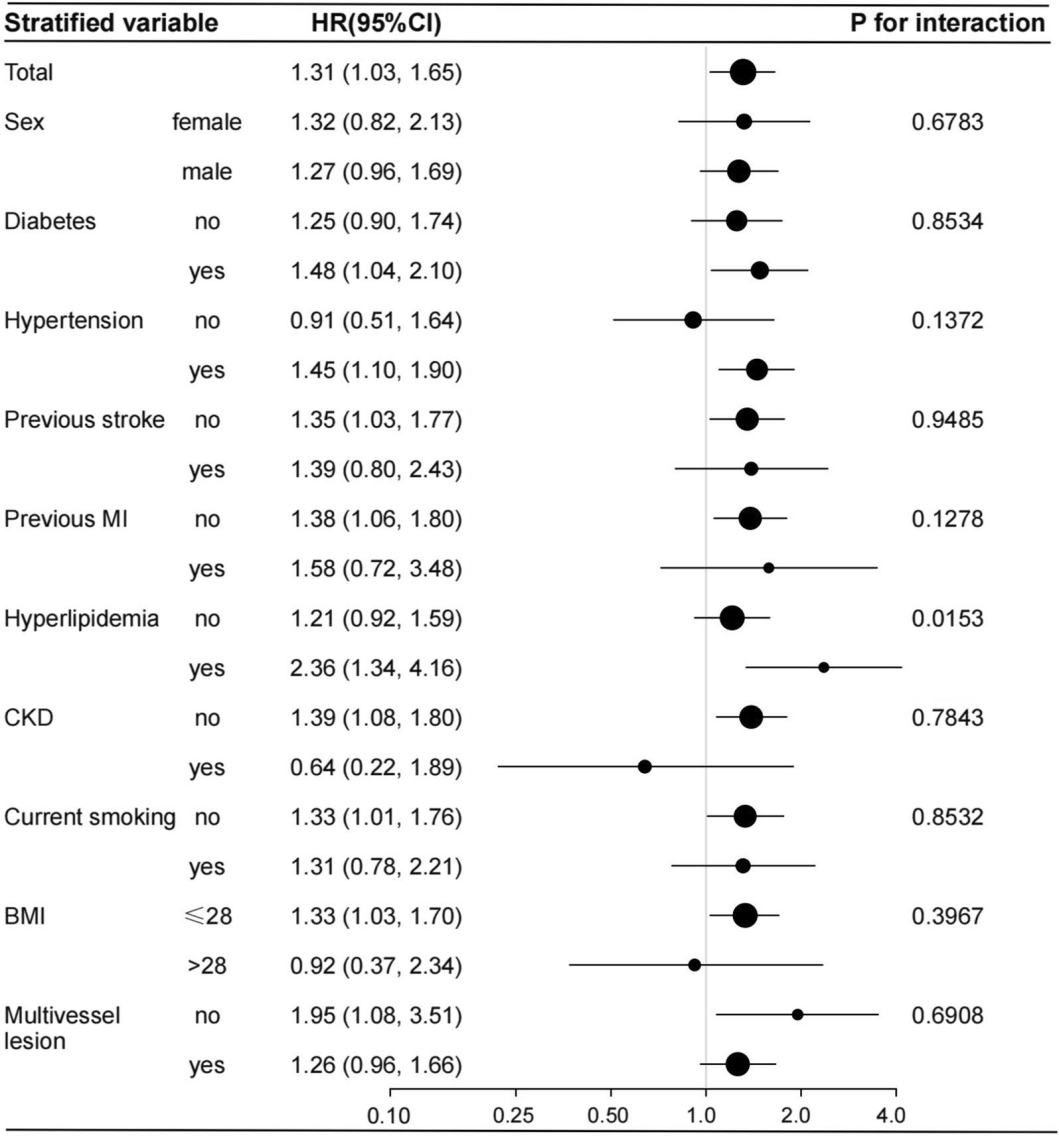
Forest plot investigating the association between the TyG index and MACE in different subgroups. Adjusted for age, gender, BMI, SBP, DBP, LVEF, Gensini score, hypertension, diabetes, hyperlipidemia, previous MI, previous stroke, CKD, current smoking, TC, LDL-C, HDL-C, eGFR, UA, aspirin, clopidogrel, statin, β-blocker, ACEI/ARB, LM lesion, multivessel lesion except for the stratified variable

Several stratified analyses were performed to further examine the relationship between the TyG index and prognosis. As shown in Fig. 2, the significantly association between TyG index and all-cause mortality in the subgroups of diabetes [HR (95% CI), 1.56 (1.05, 2.33)], those with hypertension [HR (95% CI), 1.14 (1.05, 2.12)], those without previous stoke [HR (95% CI), 1.40 (1.03, 1.90)], those without previous MI [HR (95% CI), 1.67 (1.22, 2.29)], those without hyperlipidemia [HR (95% CI), 1.54 (1.10, 2.14)], those without CKD [HR (95% CI), 1.57 (1.16, 2.11)], those no smoking [HR (95% CI), 1.65 (1.18, 2.30)], those with BMI ≤ 28 kg/m^2^ [HR (95% CI), 1.44 (1.08, 1.92)], those with multivessel disease [HR (95% CI), 1.45 (1.06, 1.98)], were consistent with the total [HR (95% CI), 1.42 (1.08, 1.86)]. Likewise, the significantly association between TyG index and MACE in the subgroups of diabetes, those with hypertension, those without previous stoke, those without previous MI, those with hyperlipidemia, those without CKD, those no smoking, those with BMI ≤ 28 kg/m^2^, those without multivessel disease, were consistent with the total. And Hyperlipidemia-stratified analyses showed a significant trend of TyG index (P for interaction = 0.0153) for MACE among the hyperlipidemia group (Fig. 3).

## Discussion

In this study, 662 elderly ACS patients were performed coronary angiography and further treatments, clinical information were collected and followed-up ten years. To the best of our knowledge, this study was the first one to explore the relationship between TyG index and the all-cause mortality and MACE in elderly ACS patients, with a focus on long-term outcomes. The main findings of our study were as follows: (1) As a reliable indicator for IR, TyG index has a significant correlation with traditional risk factors of CHD, which corresponded to previous studies [20–22]. Furthermore, we also found that except for TG and FBG, the TyG index had the highest positive correlation with the degree of coronary artery stenosis and the highest negative correlation with HDL-C; (2) TyG index had a significant effect on all-cause mortality and MACE rate. In univariate regression analysis, the all-cause mortality and MACE rate of patients increased significantly with the increase of TyG index; (3) in the fully adjusted model, the TyG index, as a continuous or categorical variable, was independently correlated with the increase of all-cause mortality and MACE rate; (4) there was a graded, positive association between TyG index and all-cause mortality and MACE; and (5) taking the TyG index into consideration was likely to have significant clinical value in early risk stratification of elderly patients with ACS.

Traditional risk factors of CHD include age, hypertension, DM, hyperlipidemia, smoking, etc. [23, 24]. Among them, DM, hyperlipidemia, and other major risk factors have been widely concerned by many scholars, but elderly ACS patients have not received enough attention. With the aging population rapidly growing worldwide, the situation of CHD in the elderly is becoming increasingly serious, and the harm and burden brought by this disease to the country, society, and family become more and more prominent. Most of the previous studies on TyG index collected middle-aged people, paid little attention to the elderly [25–27]. In addition, most of these studies have not performed stratified analyses to further validate and discuss the association between TyG index and long-term outcomes. As we all know, with the acceleration of the global aging process, the number of the elderly has increased dramatically, which has become the main goal of today's medical care. Considering the potential prognostic significance of TyG index, it is important to figure out the ability of TyG index to evaluate elderly patients with ACS. In this study, we selected elderly patients with ACS as objects, which has a good application prospect.

IR is a pathological state, which is characterized by the disorder of glucose uptake and utilization, and in the form of the decrease of insulin sensitivity. It can lead to abnormal fluctuations in blood glucose and blood lipids, manifested by increased circulating blood glucose and TC and decreased HDL-C [28]. Based on this theoretical background, TyG index is calculated according to TG and FBG, and is considered to be a reliable, convenient and inexpensive index to evaluate IR [22, 29]. The euglycemic-hyperinsulinemic clamp test is the gold standard for evaluating IR. As reported that, regardless of obesity and glucose tolerance, TyG index was negatively correlated with IR. In addition, compared with the homeostasis model assessment insulin resistance (HOMA-IR) index, TyG index showed better evaluation efficiency [30, 31]. As studies show that, TyG index is not only excellent in evaluating IR, but also closely related to coronary calcification, future cardiovascular events (including death, stroke, MI, and post-discharge revascularization), and various metabolic abnormalities, etc. and it is an independent risk factor for ACS and DES-ISR [32–35]. In this study, we found that patients with high TyG index were more likely to suffer from hypertensive, DM, stroke, hyperlipidemia and multivessel disease, which is consistent with previously reported studies [36–38]. As shown in Table 2, the TyG index had significantly positive correlation with Gensini score, and it was the first time to report the relationship between the TyG index and the degree of coronary artery stenosis in elderly ACS patients. Previous studies showed that TyG index was associated with the progression of coronary artery calcification [34]. The evaluation of noninvasive imaging such as coronary computed tomography angioplasty is limited, so the TyG index could provide a good complementary effect for coronary evaluation in this population.

With the further study of TyG index, it was found that TyG index was not only related to the traditional risk factors of CHD. Zhang et al. and Zhu et al. pointed out that if TyG index was added to the baseline risk model, the accuracy of MACE prediction in ACS and DES-ISR patients could be significantly improved [10, 39]. However, according to our 10-year follow-up study, the TyG index still has a reliable prognostic value for elderly ACS patients, especially in all-cause mortality. In addition, through subgroup analysis, we found that the association between TyG index and prognosis was stable, and this association persisted in most subgroups, including patients with DM, hypertension, stroke. It should be noted that there was a less significant interaction between TyG index and hyperlipidemia in the association between TyG index and MACE. Although the underlying mechanism is still unclear, it is necessary to consider hyperlipidemia in the prevention and management of elderly ACS patients.

Our study shows that the TyG index is a reliable indicator to evaluate the long-term prognosis of elderly patients with ACS. Although the exact mechanism is not clear, we speculate that it might be related to the fact that TyG index is a reliable index to evaluate IR. First of all, IR usually leads to the intensification of oxidative stress and inflammatory response, disturbance of glucose and lipid metabolism, activation of the renin–angiotensin–aldosterone system (RAAS), and ultimately cell damage, hypertrophy, and fibrosis occur [40, 41]. Secondly, IR might lead to excessive proliferation of vascular endothelial cells through a variety of signaling mechanisms and accelerate the progression of CHD [34]. Thirdly, as a reliable indicator to evaluate IR, TyG index is closely associated with coronary artery calcification, ISR, and various heart disease risk factors [10, 22, 34].

## Strengths and limitations

Firstly, this study is the first time to select elderly ACS patients as research subjects, and we have performed a 10-year follow-up. The results of our study have stable and reliable guiding significance. Secondly, we performed the fully adjusted analysis for as many confounders related to this research as possible and sensitivity analysis for the number, presence, and absence of cardiovascular risk factors. Thirdly, the convenience, repeatability and low cost of TyG index measurement and calculation make it have important potential to predict the prognosis of elderly ACS patients. However, there are some limitations to our study. First of all, this is a single-center cohort study with relatively small sample size, and the existing selection bias may affect the results. Further studies with large samples and multi-centers are needed to verify our results. Second, only one baseline assessment of TyG index and other risk factors was performed at admission, without taking into account fluctuations in these indicators during follow-up. Third, although we adjusted for other relevant confounders including BMI and blood lipids, we did not record energy intake and nutritional habits that might affect TG levels. Finally, all subjects in this study were selected from China, which may limit the universality of our research results on a global scale.

## Conclusion

The results of this study suggest TyG index, as a new surrogate marker for evaluating IR in clinical practice, is significantly associated with the degree of coronary artery stenosis, and is an independent predictor of long-term all-cause mortality and MACE in elderly ACS patients.

## Data Availability

The datasets used/or analyzed during the current study are available from the corresponding author on reasonable request.

## Abbreviations

CHD: Coronary heart disease
ACS: Acute coronary syndrome
IR: Insulin resistance
DM: Diabetes mellitus
TyG index: Triglyceride-glucose index
TG: Triglyceride
FBG: Fasting blood glucose
PLA: People’s Liberation Army
BMI: Body mass index
SBP: Systolic blood pressure
DBP: Diastolic blood pressure
PCI: Percutaneous coronary intervention
CABG: Coronary artery bypass grafting
LVEF: Left ventricular ejection fraction
MI: Myocardial infarction
CKD: Chronic kidney injury
TC: Total cholesterol
LDL-C: Low-density lipoprotein-cholesterol
HDL-C: High-density lipoprotein-cholesterol
eGFR: The estimated glomerular filtration rate
UA: Uric acid
ACEI: Angiotensin-converting enzyme inhibitor
ARB: Angiotensin receptor blocker
LAD: Left anterior descending artery
LCX: Left circumflex artery
LM: Left main coronary artery
RCA: Right coronary artery
Scr: Standardized creatinine
MACE: Major adverse cardiac event
AMI: Acute myocardial infarction
IQR: Interquartile range
HR: Hazard ratio
HOMA-IR: Homeostasis model assessment insulin resistance
